# Activation of AMPK by OSU53 protects spinal cord neurons from oxidative stress

**DOI:** 10.18632/oncotarget.22055

**Published:** 2017-10-23

**Authors:** Jun Xu, Liang Wu, Yiming Zhang, Huijie Gu, Zhongyue Huang, Kaifeng Zhou, Xiaofan Yin

**Affiliations:** ^1^ Department of Orthopedics, Minhang Hospital, Fudan University, Shanghai, China

**Keywords:** spinal cord injury, oxidative stress, AMPK and OSU53

## Abstract

The present study tested the potential effect of OSU53, a novel AMPK activator, against hydrogen peroxide (H2O2)-induced spinal cord neuron damages. Treatment with OSU53 attenuated H2O2-induced death and apoptosis of primary murine spinal cord neurons. OSU53 activated AMPK signaling, which is required for its actions in spinal cord neurons. The AMPK inhibitor Compound C or AMPKα1 siRNA almost abolished OSU53-mediated neuroprotection against H2O2. On the other hand, sustained-activation of AMPK by introducing the constitutive-active AMPKα1 mimicked OSU53's actions, and protected spinal cord neurons from oxidative stress. OSU53 significantly attenuated H2O2-induced reactive oxygen species production, lipid peroxidation and DNA damages in spinal cord neurons. Additionally, OSU53 increased NADPH content and heme oxygenase-1 mRNA expression in H2O2-treated spinal cord neurons. Together, we indicate that targeted-activation of AMPK by OSU53 protects spinal cord neurons from oxidative stress.

## INTRODUCTION

Oxidative stress following the spinal cord injury is a main cause of secondary damages to neurons [1–3]. The levels of different reactive oxygen species (ROS), *i.e.* superoxide anion, hydroxyl free radicals and hydrogen peroxide (H_2_O_2_), are significantly increased in the spinal cord following traumatic and non-traumatic injuries [4]. ROS production shall led to a number of harmful effects, including lipid peroxidation, protein oxidation, DNA damage, and eventually spinal cord neuronal death and apoptosis [1–4].

AMPK (AMP-activated protein kinase) is a key regulator of cellular energy [5, 6]. This heterotrimeric serine-threonine protein kinase is activated under energy crisis, which helps to maintain energy homeostasis [5, 6]. There are three AMPK subunits: α catalytic subunit along with β and γ regulatory subunits. Phosphorylation on Thr172 at the α subunit is pivotal for AMPK activation [5, 6]. Recent studies have focused the pro-survival function of AMPK. AMPK activates/inactivates its downstream effectors to promote cell survival under stress conditions [7–11]. For example, activated AMPK protects cells from oxidative stress by increasing NADPH (nicotinamide adenine dinucleotide phosphate) content [7–9]. Additionally, AMPK-activated cell autophagy could also be pro-survival [8, 10, 11].

Recently research efforts have developed a novel small-molecule AMPK activator, namely OSU53 [12]. Unlike other known AMPK activators (i.e. AICAR [13] and aspirin [14]), OSU53 binds directly to the α subunit, which activates AMPK with an excellent EC-50 (around 1–10 μM) [15–17]. Meanwhile, OSU53 displays fine oral bioavailability, which is delivered in its metabolically active state (no further modification is needed) [15–17]. The current study tested its potential effect in spinal cord neurons against oxidative stress.

## RESULTS

### OSU53 protects murine spinal cord neurons from H_2_O_2_

**Figure 1:**
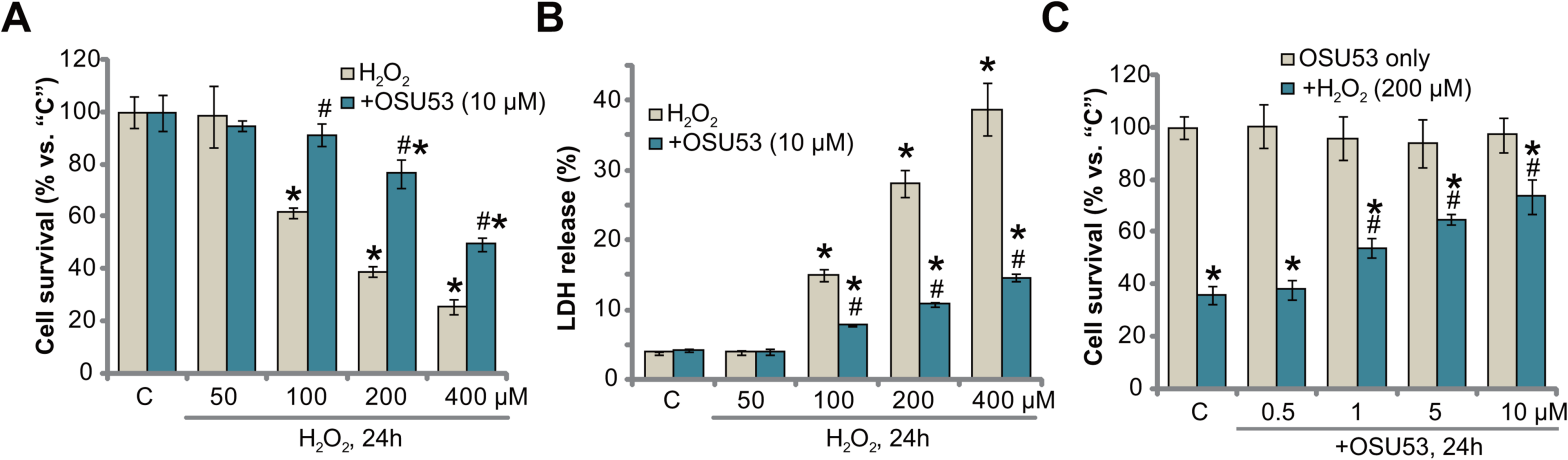
OSU53 protects murine spinal cord neurons from H_2_O_2_ Primary cultured murine spinal cord neurons were treated with hydrogen peroxide (H_2_O_2_, 50–400 μM), or plus employed concentration of OSU53, for 24 hours; Neuronal survival and death were tested by CCK-8 assay (**A** and **C**) and LDH release assay (**B**), respectively. The data were presented as mean ± standard deviation (SD) (Same for all Figures). For each assay, *n* = 5. “C” stands for untreated control group (Same for all Figures). ^*^p < 0.05 vs. “C”. ^#^*p* < 0.05 vs. H_2_O_2_ only group. Experiments in this figure were repeated five times, and similar results were obtained.

To mimic oxidative stress *in vitro*, primary cultured murine spinal cord neurons were treated with hydrogen peroxide (H_2_O_2_) at different concentrations, from 50–400 μM. Cell Counting Kit-8 (CCK-8) assay results in Figure 1A demonstrated that H_2_O_2_ dose-dependently inhibited survival of the murine spinal cord neurons. The CCK-8 optic density (OD) value was decreased significantly following 100–400 μM of H_2_O_2_ treatment (at 24 hours, Figure 1A). Significantly, co-treatment with OSU53 (10 μM), the novel AMPK activator [18, 19], significantly inhibited spinal cord neuron viability (CCK-8 OD) reduction (Figure 1A). For instance, the CCK-8 OD of the neurons decreased to 38.93 ± 2.18% of control level after H_2_O_2_ (200 μM, 24 hours) treatment. Following OSU53 (10 μM) co-treatment, its level recovered to 76.47 ± 5.30% of control level (Figure 1A).

Decreased cell survival could be due to cell death. The release of lactate dehydrogenase (LDH) to the conditional medium is the well-established marker of cell death. We here demonstrated that treatment with 100–400 μM of H_2_O_2_ significantly increased medium LDH release, suggesting spinal cord neuronal death (Figure 1B). Such cytotoxic effect was again largely attenuated with co-treatment of OSU53 (10 μM) (Figure 1B). The potential dose response of OSU53 was also tested. As demonstrated in Figure 1C, co-treatment with OSU53 at 1–10 μM significantly attenuated H_2_O_2_ (200 μM)-induced viability reduction of the spinal cord neurons. At a lower concentration (0.5 μM), this novel AMPK activator was in-effective against H_2_O_2_ (Figure 1C), thus confirming a dose-dependent response. Collectively, these results suggest that OSU53 protects murine spinal cord neurons from H_2_O_2_.

### OSU53 inhibits H_2_O_2_-induced spinal cord neuron apoptosis

**Figure 2:**
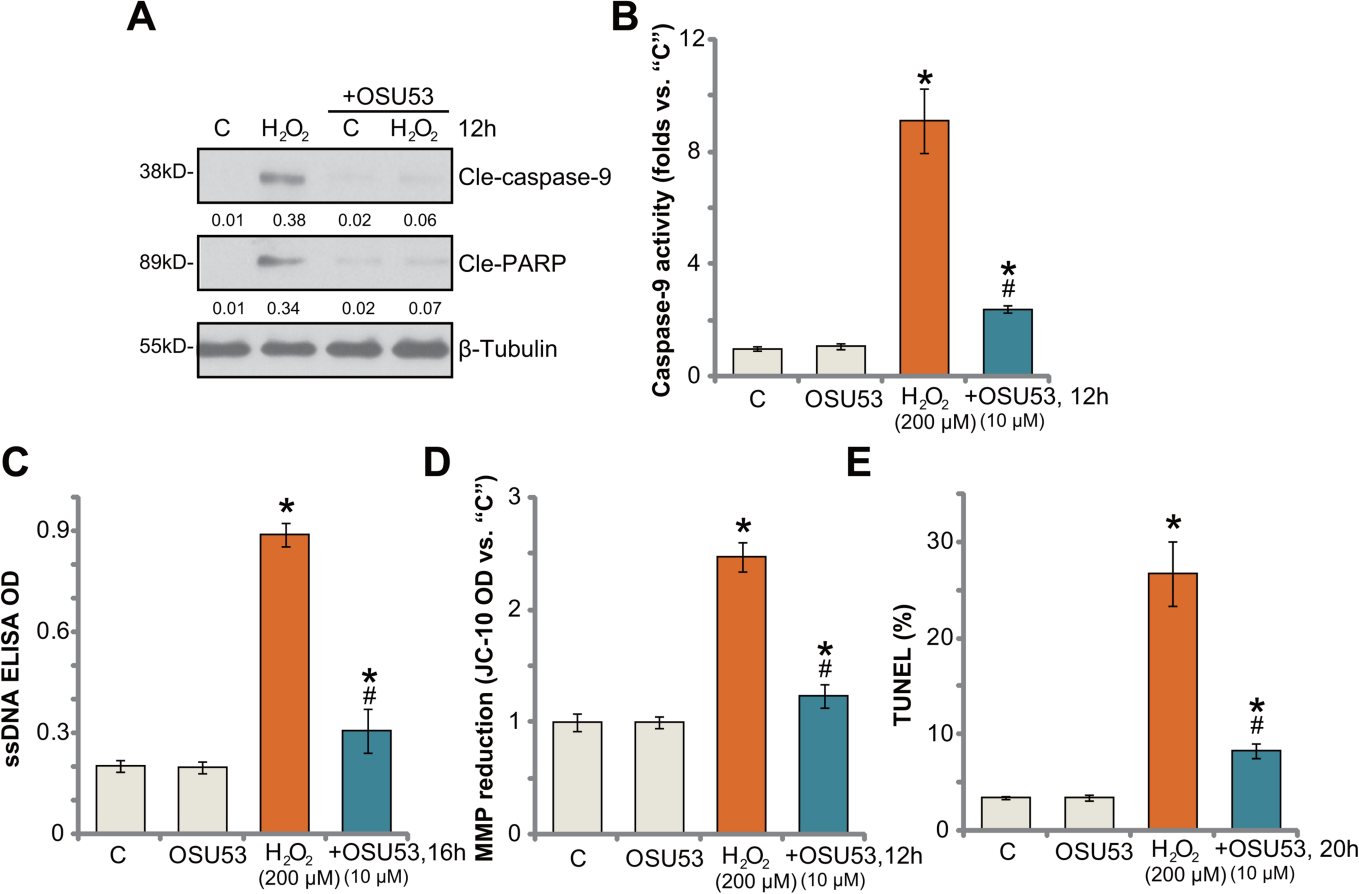
OSU53 inhibits H_2_O_2_-induced spinal cord neuron apoptosis Primary cultured murine spinal cord neurons were treated with hydrogen peroxide (H_2_O_2_, 200 μM), or plus OSU53 (10 μM) for indicated time; Cell apoptosis was tested by the assays mentioned in the text (**A–E**). For each assay, *n* = 5. ^*^p < 0.05 vs. “C”. ^#^*p* < 0.05 vs. H_2_O_2_ only group. Experiments in this figure were repeated three times, and similar results were obtained.

Next, the potential effect of OSU53 on H_2_O_2_-induced neuronal apoptosis was analyzed. As shown in Figure 2A, treatment with H_2_O_2_ (200 μM) in cultured murine spinal cord neurons induced cleavage of both caspase-9 and PARP [poly (ADP-ribose) polymerase]. Meanwhile, the caspase-9 activity was also increased in H_2_O_2_-treated neurons (Figure 2B). Such actions by H_2_O_2_ were largely attenuated with co-treatment of OSU53 (Figure 2A–2B). Additionally, H_2_O_2_ stimulation in the neurons also induced production of single strand DNA (“ssDNA”) (Figure 2C), mitochondrial depolarization (Figure 2D, JC-1 OD increase [20]) and TUNEL (terminal transferase uridyl nick end labeling) nuclei increase (Figure 2E), which all largely attenuated by OSU53. Therefore, these results suggest that OSU53 inhibits H_2_O_2_-induced spinal cord neuronal apoptosis.

### AMPK activation mediates OSU53-induced neuroprotection against H_2_O_2_

**Figure 3:**
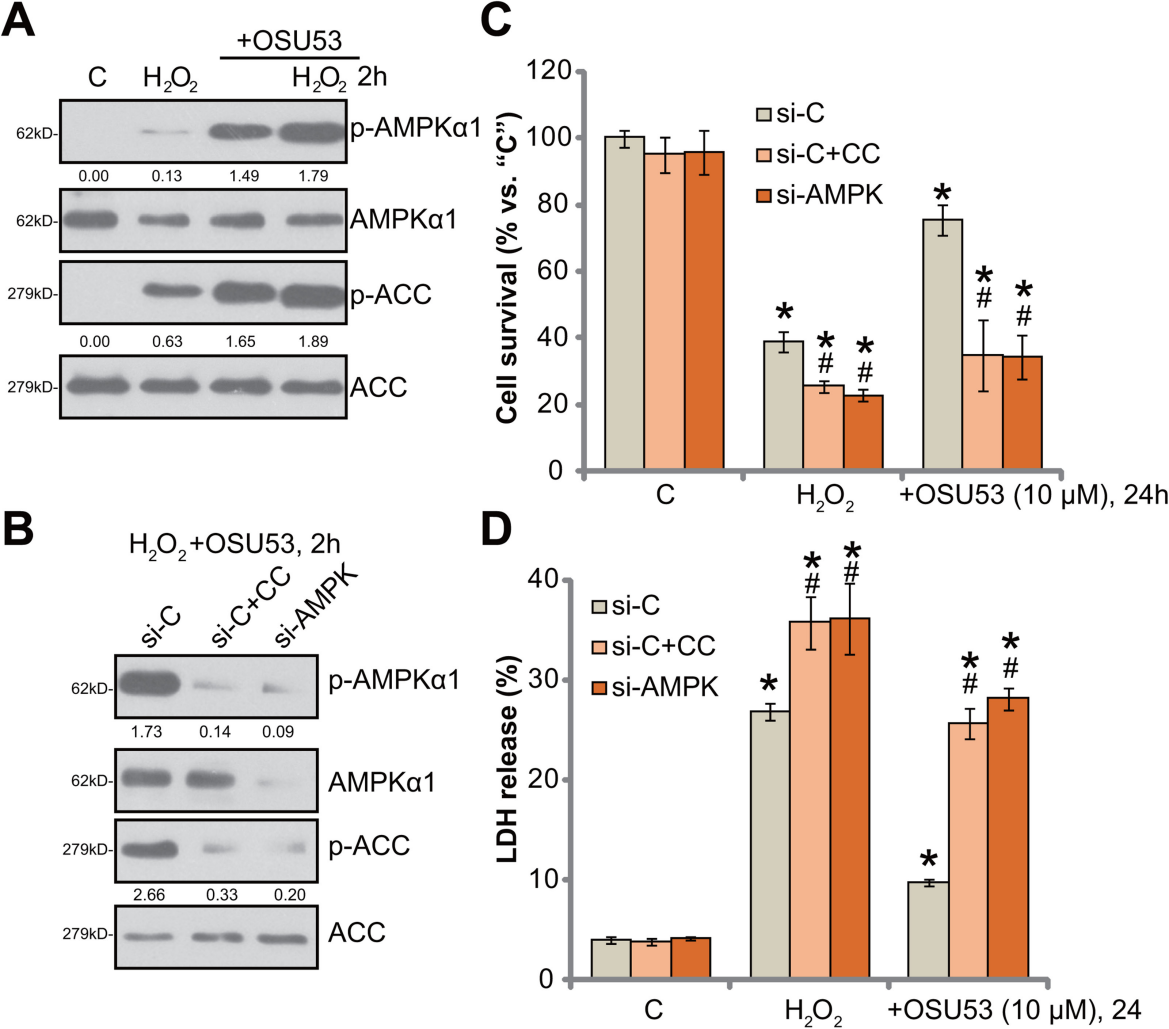
AMPK activation mediates OSU53-induced neuroprotection against H_2_O_2_ Primary cultured murine spinal cord neurons were treated with hydrogen peroxide (H_2_O_2_, 200 μM), or plus OSU53 (10 μM) for 2 hours, expressions of total and phosphorylated (“p-”) AMPKα1 and ACC were shown (**A**). Phosphorylations AMPKα1 (vs. total AMPKα1) and ACC (vs. total ACC) were quantified (A). Primary cultured murine spinal cord neurons, were pretreated with the AMPK inhibitor Compound C (10 μM, for 1 hour) or AMPKα1 siRNA (200 nM, 2 rounds, total 24 hours), followed by hydrogen peroxide (H_2_O_2_, 200 μM), or plus OSU53 (10 μM) treatment for employed time; Expressions of listed proteins were shown (**B**), phosphorylations of AMPKα1 (vs. β-Tubulin) and ACC (vs. total ACC) were quantified (B); Neuronal survival (CCK-8 assay, (**C**) and death (LDH release assay, (**D**) were also tested. “si-C” stands for non-sense scramble siRNA (B–D). For each assay, *n* = 5. ^*^p < 0.05 vs. “C”. ^#^*p* < 0.05 vs. “si-C” group. Experiments in this figure were repeated three times, and similar results were obtained.

OSU53 is the novel AMPK activator [15, 16], the requirement of AMPK in OSU53-meidated neuroprotection was then tested. Western blotting assay results in Figure 3A confirmed that OSU53 treatment induced significant AMPK activation in cultured murine spinal cord neurons. AMPK activation was reflected by phosphorylations (“p-”) of AMPKα1 (at Thr-172) and acetyl-CoA carboxylase (ACC, Ser-79), the latter is the AMPK's major downstream substrate [21, 22]. Intriguingly, H_2_O_2_ also induced minor AMPK activation, or AMPKα1/ACC phosphorylations (Figure 3A), which was largely enhanced with OSU53 co-treatment (Figure 3A).

To study the link between AMPK activation and OSU53-mediated neuroprotection, pharmacological and genetic strategies were employed to block AMPK activation. Compound C is a well-established AMPK inhibitor [23]. We here demonstrated that compound C blocked OSU53-induced AMPK activation, or AMPKα1/ACC phosphorylations, in H_2_O_2_-treated spinal cord neurons (Figure 3B). Further, siRNA strategy was utilized to knockdown AMPKα1. The applied AMPKα siRNA (Santa Cruz Biotech Co) efficiently downregulated AMPKα1 in the spine cord neurons (Figure 3B). Subsequently, OSU53-induced AMPK activation was inhibited (Figure 3B). Remarkably, OSU53-mediated neuroprotection against H_2_O_2_ was almost abolished by Compound C or AMPKα1 siRNA (Figure 3C and 3D). OSU53 was ineffective to protect spine cord neurons when AMPK was inhibited or silenced (Figure 3C and 3D). These results suggest that AMPK activation is required for OSU53-mediated neuroprotection. It should be noted that Compound C or AMPKα1 siRNA also exacerbated H_2_O_2_-induced cytotoxicity (Figure 3C and 3D), arguing that AMPK activation by H_2_O_2_ itself is also neuroprotective.

### Constitutive activation of AMPK protects spinal cord neurons from H_2_O_2_

**Figure 4:**
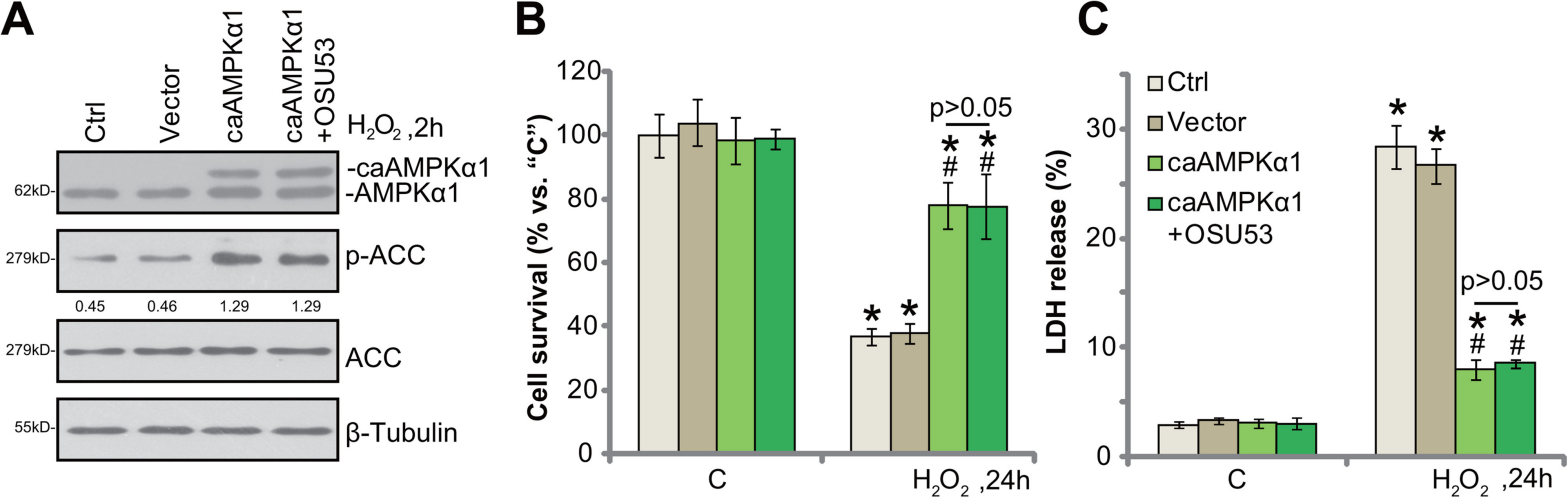
Constitutive activation of AMPK protects spinal cord neurons from H_2_O_2_ The primary cultured murine spinal cord neurons, expressing the constitutive-active AMPKα1 (T172D, “caAMPKα1”, Flag-tagged) or empty vector (pSuper-puro-Flag, “Vector”), as well as the control neurons (“Ctrl”) were treated with hydrogen peroxide (H_2_O_2_, 200 μM), or plus OSU53 (10 μM) for employed time, total and phosphorylated (“p”) AMPKα1 and ACC were shown (**A**), p-ACC (vs. total ACC) were quantified (A); Neuronal survival (CCK-8 assay, (**B**) and death (LDH release assay, (**C**) were also tested. For each assay, *n* = 5. ^*^p < 0.05 vs. “C”. ^#^*p* < 0.05 vs. “Ctrl” cells. Experiments in this figure were repeated three times, and similar results were obtained.

Based on the above-mentioned results, we can speculate that forced-activation of AMPK shall also protect spinal cord neurons from oxidative stress. To test this hypothesis, a constitutive-active AMPKα1 (T172D, caAMPKα1, by Dr. Wang [9]) was transfected to the spinal cord neurons. The caAMPKα1 construct had the constitutive phosphorylation of AMPKα1 at Thr-172, leading to sustained AMPK activation [9, 24]. Western blotting assay results confirmed expression of caAMPKα1 (tagged with Flag) in the spine cord neurons (Figure 4A). AMPK activation, tested by p-ACC, was significantly increased in caAMPKα1-expressing neurons, even in the presence of H_2_O_2_ (Figure 4A). Remarkably, forced-expression of caAMPKα1 protected the spinal cord neurons from H_2_O_2_ (Figure 4B and 4C). Thus, sustained activation of AMPK by caAMPKα1 mimicked OSU53's actions and protected neurons from oxidative stress. It should be noted that adding OSU53 in caAMPKα1-expressing neurons failed to further inhibit H_2_O_2_-induce damages (Figure 4B and 4C). Thus, OSU53 was ineffective in AMPK-pre-activated neurons, again confirming that AMPK activation is required for OSU53-mediated neuroprotection against H_2_O_2_.

### OSU53 inhibits H_2_O_2_-induced oxidative stress in spinal cord neurons

**Figure 5:**
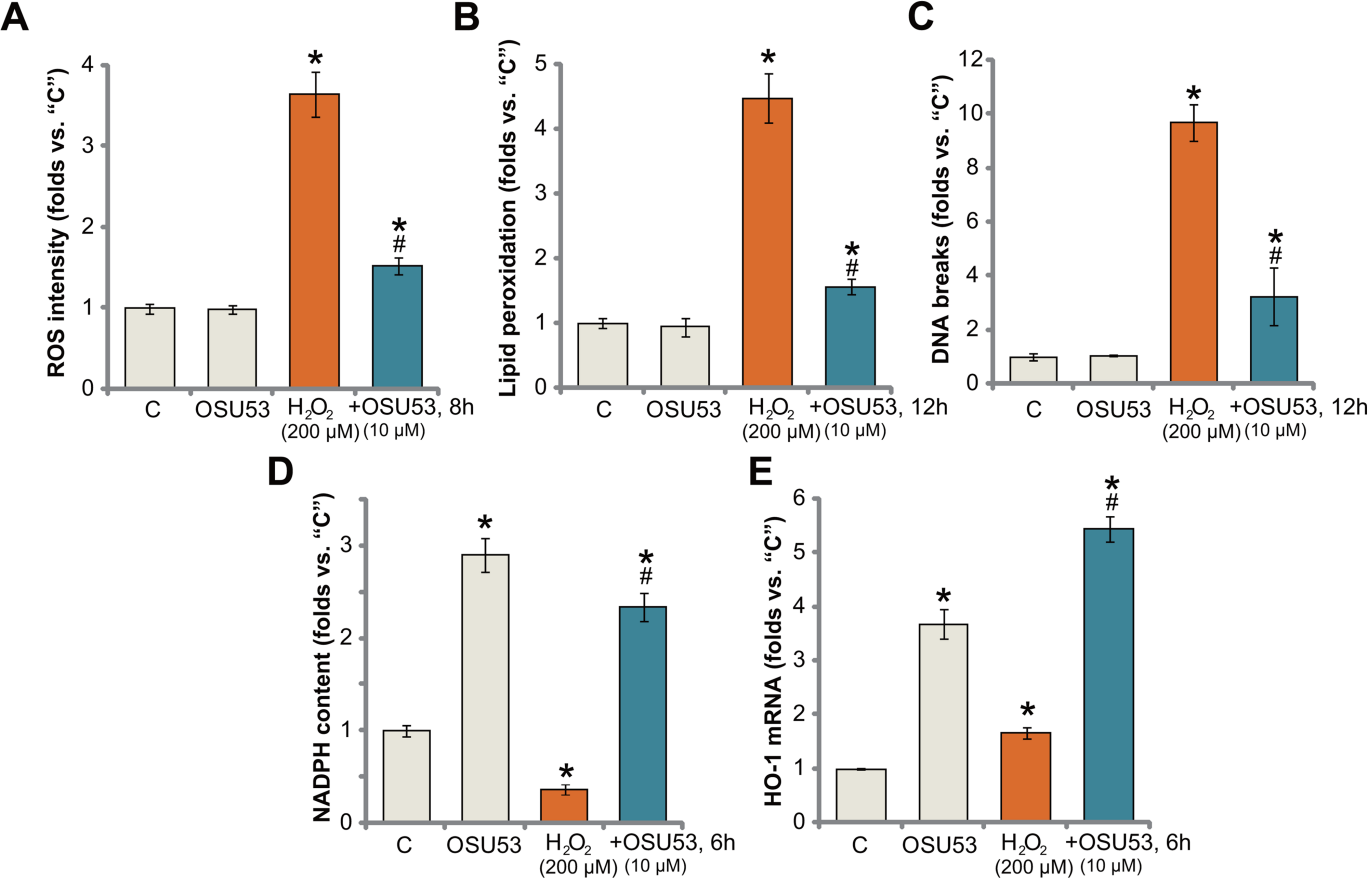
OSU53 inhibits H_2_O_2_-induced oxidative stress in spinal cord neurons Primary cultured murine spinal cord neurons were treated with hydrogen peroxide (H_2_O_2_, 200 μM), or plus OSU53 (10 μM) for indicated time; Relative ROS production (**A**), lipid peroxidation content (**B**) and DNA damages (**C**) were tested by the described methods; Relative NADPH content (**D**) and *HO-1 mRNA* expression (**E**) were also tested. For each assay, *n* = 5. ^*^p < 0.05 vs. “C”. ^#^*p* < 0.05 vs. H_2_O_2_ only group. Experiments in this figure were repeated three times, and similar results were obtained.

Recent studies have confirmed a cytoprotective function of AMPK under various stress conditions. In particularly, activated AMPK is capable of fighting oxidative stress [25, 26]. AMPK inhibits reactive oxygen species (ROS) production, thus protecting cells from oxidative stress [27]. Here, we show that treatment with OSU53 also significantly attenuated H_2_O_2_-induced ROS production (DCFH-DA fluorescent dye intensity) in spinal cord neurons (Figure 5A). Consequently, H_2_O_2_-induced lipid peroxidation (Figure 5B) and DNA damages (tested by γ-H2AX percentage, Figure 5C) were also dramatically alleviated by OSU53 pre-treatment. These results suggest that targeted-activation of AMPK by OSU53 significantly inhibited H_2_O_2_-oxidative stress in spinal cord neurons, which could be the major reason to explain the neuroprotection activity by this novel AMPK activator.

There are proposed mechanisms responsible for AMPK-mediated anti-oxidant activity. As discussed, AMPK maintains NADPH homeostasis to promote cell survival under stress conditions [7–9, 19, 25]. AMPK is also positively involved in activation of Nrf2 (nuclear factor erythroid 2-related factor 2) [28, 29], the latter is a major anti-oxidant transcription factor responsible for transcription of heme oxygenase (HO)-1 and many other key anti-oxidant genes [30–34]. Here, we show that OSU53 treatment also induced NADPH synthesis in the spinal cord neurons (Figure 5D). More importantly, although H_2_O_2_ decreased NADPH content in the neurons (Figure 5D), after co-treatment OSU53, its level was restored (Figure 5D). Further studies showed that OSU53 treatment in spinal cord neurons also induced transcription of *HO-1 mRNA* (Figure 5E). Additionally, H_2_O_2_-induced *HO-1 mRNA* expression was further enhanced with OSU53 co-treatment (Figure 5E). These results suggest that OSU53-mediated anti-oxidant response could be due to AMPK-dependent NADPH production and/or Nrf2-HO-1 signaling activation.

## DISCUSSION

In the current study, we show that OSU53 activated AMPK signaling, which is required for its activity in spinal cord neurons. Compound C, the AMPK inhibitor, or AMPKα1 siRNA almost abolished OSU53-mediated neuroprotection against H_2_O_2_. On the other hand, sustained-activation of AMPK by introducing the ca-AMPKα1 (T172D) mimicked OSU53's actions and protected spinal cord neurons from oxidative stress. More importantly, adding OSU53 was ineffective in ca-AMPKα1-expressing neurons against H_2_O_2_. Therefore, targeted-activation of AMPK by OSU53 protects the spinal cord neurons from oxidative stress.

A number of recent studies have proposed the anti-oxidant function of activated AMPK under stress conditions [7, 8, 35, 36]. AMPK was initially found to be vital for maintaining cellular NADPH content [7]. AMPK phosphorylates and in-activates its major substrate protein ACC, thus reducing NADPH consumption [7]. Additionally, AMPK activation shall promote NADPH synthesis and production [25, 37, 38] through the mechanisms involving fatty-acid oxidation [7]. The study by Guo's group demonstrated that targeted-activation of AMPK by Compound 13, the α1 specific AMPK activator [39, 40], increased NADPH production to inhibit dexamethasone (Dex)-induced oxidative stress, thus offering protection to human osteoblastic cells [36]. Similarly, miR-135b downregulated Ppm1e, the AMPK phosphatase [41–43], to activate AMPK and protect cells from oxidative stress [25]. Here, we show that targeted-activation of AMPK by OSU53 also increased NADPH content in the spinal cord neurons. More importantly, co-treatment with the novel AMPK activator also restored NADPH content in H_2_O_2_-treated neurons. Thus, AMPK-mediated NADPH axis could be one key mechanism responsible for OSU53-mediated neuroprotection against oxidative stress.

Nrf2 is arguably one of the most important anti-oxidant signaling in mammalian cells [44–46]. As a transcription factor, Nrf2 binds to antioxidant-responsive element (ARE), dictating transcription of multiple key anti-oxidative enzymes [44–46], including HO-1, NAD(P)H quinone oxidoreductase 1 (NQO1), γ-glutamyl cysteine ligase catalytic subunit (GCLC) [47], along with many others [44–46]. Recent studies have explored the potential cross-talk between AMPK and Nrf2/HO-1 signalings. It has been shown that activated AMPK could boost the Nrf2/HO-1 signaling axis involving unfolded protein response [28]. Meanwhile, Joo *et al.,* showed that AMPK directly phosphorylates Nrf2 at the Ser-550, which shall promote Nrf2 nuclear accumulation and following HO-1 transcription with the help from GSK3 inhibition [29]. Therefore, AMPK-boosted Nrf2/HO-1 activation could be another key mechanism underlying its anti-oxidant activity. Indeed, we show that *HO-1 mRNA* level was significantly increased in OSU53-treated spinal cord neurons. The detailed mechanisms, of course, warrant further characterizations.

It is estimated that there will over 150,000–200,000 cases of traumatic spinal cord injury annually due to accidents and violence [1, 2, 4]. The clinical and bench results have indicated that severity of neurological deficit is not solely dependent on the degree of the mechanical insults [1, 2, 4]. It is, interestingly, very closely associated with secondary damaging factors, including ischaemia, oedema, inflammatory response and most importantly oxidative stress [1, 2, 4]. The results of this study suggest that targeted-activation of AMPK by OSU53 efficiently protects the spinal cord neurons from oxidative stress, suggesting that this novel AMPK activator might be further tested in animal and clinical models of spinal cord injury.

## MATERIALS AND METHODS

### Reagents and antibodies

OSU53 was provided by Dr. Cui [19], which was dissolved in dimethyl sulfoxide (DMSO). H_2_O_2_ and compound C were obtained from Sigma Aldrich Co. (Shanghai, China). Antibodies of p-AMPKα1 (Thr172, #2531, 1: 1000), AMPKα (#2532, 1: 2000), acetyl-CoA Carboxylase (ACC, #3662, 1: 2000), p-ACC (Ser79, #3661, 1: 1000) and (β-) Tubulin (#2146, 1: 20,000) were purchased from Cell Signaling Technology (Beverly, MA). The cell culture reagents were provided by GIBCO (Rockville, MD).

### Murine spinal cord neurons

The detailed protocol of culture of primary murine spinal cord neurons was described in detail previously [48]. In brief, the spinal cord of C57BL/6J mouse embryos (E13-14) were initially plated at 200,000 cells/mL onto the tissue-culture plate, pre-coated with poly-l-lysine (Sigma). The neuronal feeding medium, 90% MEM, 5% horse serum, 5% fetal bovine serum (FBS) (GIBCO), and a mixture of nutrient supplements [48], was replaced with feeding medium every 2–3 days.

### Survival assay

The Cell Counting Kit-8 (CCK-8) kit, purchased from the Dojindo Laboratories (Kumamoto, Japan), was employed to examine the viability of spinal cord neurons. CCK-8 optic density (OD, at 450 nm) of treatment group was always normalized to that of untreated control group.

### Death assay

The release of lactate dehydrogenase (LDH) to the conditional medium is a well-established marker of neuronal cell death, which was tested by a commercial available two-step LDH assay kit (Takara, Tokyo, Japan) [49].

### Enzyme-linked immunosorbent assay (ELISA) of cell apoptosis

The increased production of single stream DNA (ssDNA) is a characteristic marker of cell apoptosis [50]. ssDNA content in spinal cord neurons was tested by a commercial available ssDNA ELISA kit (Chemicon International, Temecula, CA). ELISA OD (at 405 nm) was recorded to reflect cell apoptosis.

### TUNEL assay

Spinal cord neurons with described treatment were stained with TUNEL and DAPI florescence dyes (All purchased from Sigma). TUNEL staining ratio (vs. DAPI nuclei number) was recorded.

### Caspase-9 activity assay and mitochondrial depolarization assay

The caspase-9 activity assay by the caspase-Glo 9 kit (Promega, Shanghai, China) and mitochondrial membrane potential (MMP) reduction assay by the JC-1 dye (Invitrogen, Shanghai, China) were described in detail in previous studies [20, 51].

### Western blotting assay

Spinal cord neurons were first trypsinized and washed. Neurons were then incubated with the cell lysis buffer (Biyuntian, Wuxi, China). Protein concentration was determined using the Bio-Rad protein assay kit (Shanghai, China). For each single treatment, exact 30–40 μg total lysate proteins (per lane) were separated by the SDS-PAGE gels (7.5–10%) [52, 53], which were then transferred to the PVDF blots (Millipore, Suzhou, China). After blocking, the blots were incubated with designated primary and secondary antibodies. To visualize the targeted bands, the enhanced chemiluminescence (ECL) reagents (Pierce) were added, and X-Ray film development was employed.

### qRT-PCR assay

Spinal cord neurons were incubated with TRIzol reagents (Invitrogen) to achieve total cellular RNA. Quantitative Real-time PCR (“qRT-PCR”) assay was performed BY the SYBR green kit on the ABI-7600 fast PCR system (Applied Biosystems, Shanghai, China) [33]. mRNA primers for murine *HO-1* and *GAPDH* were described previously [47, 54]. *GAPDH mRNA* was always tested as the internal control and reference gene. The 2^-ΔΔCt^ method was employed to calculate relative *HO-1 mRNA* expression (vs. *GAPDH mRNA)*.

### siRNA

The siRNA of murine AMPKα1 was purchased from Santa Cruz Biotech. The AMPKα1 siRNA or the scramble control siRNA (200 nM, 12 hours × two rounds) transfection to spinal cord neurons was performed using the Lipofectamine 2000 reagent (Invitrogen). Afterwards, AMPKα1 knockdown in the resulting neurons was verified by Western blotting assay.

### AMPKα1 mutation

The constitutive-active AMPKα1 (T172D, caAMPKα1) was provided by Dr. Wang at Soochow University [9]. The spinal cord neurons were seeded onto 6-well plates at 50–60% confluence. The caAMPKα1 cDNA (0.25 μg per well) was transfected to neurons via the Lipofectamine 2000 protocol [55]. Transfection efficiency was always verified via Western blotting assay.

### NADPH assay

The NADPH content was tested via the methods described previously [8, 56]. Spinal cord neurons with the described treatment were trypsinized and washed with PBS, and incubated with the lysis buffer (Biyuntian, Wuxi, China). The protein lysates were then incubated with NADP-cycling buffer along with glucose-6-phosphate dehydrogenase (G6PD, Sigma) at 60 °C for 30 min [8], which was followed by addition of glucose 6-phosphate (G6P, Sigma). Thereafter, G6P's absorbance change at 570 nm was tested every 30 seconds for 4 min. NADPH content was then recorded and normalized to that of untreated control group [56].

### Reactive oxygen species (ROS) assay

The dichlorofluorescin diacetate (DCFH-DA) fluorescent dye (Invitrogen) was employed to quantify cellular ROS intensity [25]. In brief, 1 μM of DCFH-DA were added to the cultured spinal cord neurons for 45 min. Neurons were then washedS, and subjected to examination of fluorescence intensity under a Fluorescence Microplate Reader (Synergy 2, BioTek, Winooski, VT). ROS intensity OD was expressed as fold change of untreated control group.

### Lipid peroxidation assay

The thiobarbituric acid reactive substances (TBAR) assay was employed to test the cellular lipid peroxidation intensity. The detailed protocol was described previously [57, 58].

### DNA damage assay

In the current study, γ-H2AX intensity was tested to reflect cellular DNA damages [59]. In short, spinal cord neurons with the applied treatment were trypsinized and fixed. Afterwards, anti-γ-H2AX antibody (Cellular Signaling Tech) was added to the neurons for additional 4 hours. The FITC-conjugated secondary antibody was then added to theγ-H2AX-labbled neurons, which were then subjected to FACS assay. γ-H2AX percentage was recorded [59].

### Statistical analysis

The results were expressed as mean ± standard deviation (SD). The statistical analysis among different groups was done using one-way ANOVA with Scheffe's test. Experiments were repeated at least three times and consistent results were always obtained.
